# Letter from S. G. Armor, M. D.

**Published:** 1848-02

**Authors:** S. G. Armor

**Affiliations:** New York


ARTICLE II.

### LETTER FROM S. G. ARMOR, M. D.

New York, Feb. 8, 1848.

Dr. Brainard:

*Dear Sir*,—In my last letter I gave you the chemical composition of the chloroform, an article recently introduced to the profession, and one which promises to be of much service in rendering patients insensible even to the most terrible surgical operations. It is extensively, but at the same time judiciously and cautiously used here, and, so far as I learn, with very satisfactory results. It appears to have entirely superceded the sulph. ether.

Another article, new to the profession generally, but of which you have doubtless seen an account in some of the recent journals, is the *gutta percha*. It has been recently introduced from Europe, chiefly as an article of trade, and is seized upon by the *surgical* portion of the profession here with great avidity. It is a substance somewhat analogous, in its physical properties and appearance, to gum elastic or India rubber, and is said to be admirably adapted to the treatment of certain forms of fracture, especially fractures of bones occurring near their articulations, such as the *condyles* of the humerus. It is well known that these are a class of fractures at all times difficult to manage. A leading indication in their treatment, being a flexed position of the arm, a dressing which can be easily removed and readily changed, in order that a different flexion and a sufficient amount of passive motion may be kept up from time to time, thereby preventing anchylosis of the joint, is, to say the least, a matter of great convenience. This substance would appear to answer this purpose in an eminent degree, from the fact that it posesses the property, when heated for a few minutes in warm water, of entire *plasticity*; it can be readily moulded to any shape; and when cool, *preserves its shape*, forming a firm caste, yet sufficiently elastic to render it a comfortable and safe dressing. It is highly spoken of by the profession here who have tried it, and whose extensive experience in the treatment of fractures has rendered them familiar with the difficulties which are often encountered.

The Medical Colleges of the city have both very fair classes, although not much increased in numbers, if any, from last year. The University numbers some 400, and the College of Physicians and Surgeons about 190 or 200 students. Eastern medical schools do not appear to be increasing in the same ratio with those of the west. Advantages of instruction being equal, many western students prefer western institutions where medicine is *taught* as it is *practised*, and, indeed, so much do the peculiar epidemic fevers of the Mississippi valley become complicated with, and give character to many chronic diesases of the south and west, that it is not without *reason* students seek schools of medical instruction in that region. To one, familiar with the diseases which present themselves for clinical instruction, in the west and east, this is seen in a very striking view. While typhus and typhoid—pneumonia, phthisis, scrofula and syphilis, with all its loathsome concomitants, are constantly presented for instruction and treatment here; our quotidians, tertians and quartans—"hob-nail" livers and "ague-cake" of the west, with all their pathological relations, are rarely met with. Almost daily in attendance, at the City Hospital, since my arrival in the city, and at the clinics connected with each of the Medical Colleges, I have seen but *one* patient from the miasmatic regions of the west. He, by some means, found his way here from our State of Illinois, and such cases being rare, and constituting a pleasant variety for clinical instruction, he was jocosely presented to the class by the good natured Professor, as "one of the men we *read* about," and his disease, one of *secondary* character, was thought to be one of much interest, as originally growing out of an attack of fever, peculiar to the country, from which he came. For the general student of medicine, however, New York affords many advantages. The city and its environs, containing a population of almost half a million, with its crowded hospitals, infirmaries and dispensaries, furnish abundant opportunities for the acquisition of professional knowledge. Indeed, so far as relates to the unfortunate poor, who are thrown into these institutions of charity, New York may well claim a sad pre-eminence over all her neighboring cities.

I am pleased with the spirit manifested by the profession of New York. Perhaps at no time in the history of this Metropolis was medicine cultivated *as a science*, to the same extent it is at present. And this remark will, doubtless, apply to the profession, generally, of this country. It is *not true* that our science is *stationary*. At no time were more united and successful efforts made to advance the science of medicine This advance is seen in the increased attention given to the study of *pathology* ; the study of animal and organic chemistry, and therapeutical remedies, in their direct relations to *pathological conditions* ; greater accuracy and precision in statement of *facts* from premises ; a more varied scope of enquiry, and an *augmented spirit of original research*, with less reference to abstract and speculative theory. These things, faithfully and energetically carried out, must and will give stability and character to our medical literature. Respectfully, yours,

S. G. Armor.

